# Novel indole and quinoline alkaloids from *Melodinus yunnanensis*

**DOI:** 10.1007/s13659-011-0001-0

**Published:** 2011-09-02

**Authors:** Xiang-Hai Cai, Yan Li, Jia Su, Ya-Ping Liu, Xiao-Ning Li, Xiao-Dong Luo

**Affiliations:** State Key Laboratory of Phytochemistry and Plant Resources in West China, Kunming Institute of Botany, Chinese Academy of Sciences, Kunming, 650201 China

**Keywords:** spiro-indolone, quinoline, meloyunine, *Melodinus yunnanensis*

## Abstract

6/7-Seco rearranged spiro-indolone alkaloids, meloyunines A (**1**) and B (**2**) and a monoterpenoid quinoline alkaloid meloyunine C (**3**) together with its possible intermediate 14,15-dehydromelohenine B (**4**), and their precursor Δ^14^-vincamenine (**5**) were isolated from *Melodinus yunnanensis*. All structures were elucidated based on NMR, FTIR, UV, and MS spectroscopic data. The isolation of monoterpenoid indole, quinoline, and its immediate from the same plant chemically supported the biosynthesis of quinoline from indole. Compound **2** was cytotoxic against several human cancer cell lines.

**Electronic Supplementary Material:**

Supplementary material is available for this article at 10.1007/s13659-011-0001-0 and is accessible for authorized users.

## Introduction

Monoterpenoid indole alkaloids originate from the condensation of tryptophan with secologanin to produce strictosidine, which further alters by rearrangement to yield a dozen subgroups.[1] Some of the remarkable quinoline alkaloids, such as quinine and camptothecin which are well known for their antimalarial and anticancer properties, respectively, have been proposed to arise by rearrangement of monoterpenoid indole alkaloids. In a possible route for quinine biosynthesis, the cleavage of a $N_1$-$C_2$ bond in the indole heterocyclic ring could generate new amine and keto functions. A new quinoline heterocycle would then be formed by combining this N-1 amine with a C-5 aldehyde produced by a tryptamine side-chain cleavage, producing cinchonidinone.[2] Unlike quinine, the proposed biosynthesis of camptothecin includes a $C_2$–$C_7$ double bond oxidation to yield two carbonyls and an aldol-type condensation between $C_2$ and $C_6$ to form a quinoline ring. An *in vivo* tracer experiment has supported the prediction that the quinoline moiety originates from tryptophan.[3] Also, melodinine B, a possible key intermediate of indole to quinoline alkaloids, has been reported.[4] Plants of the genus *Melodinus* have been shown to be good sources of monoterpenoid indole and quinoline alkaloids.[5] During our search for novel and bioactive monoterpenoid indole alkaloids from the family Apocynaceae, some representative skeletons and cytotoxic compounds were reported from the genera *Alstonia*[6] and *Melodinus*.[4,7] This paper describes skeletons and cytotoxic compounds were reported from the genera *Alstonia*[6] and *Melodinus*.[4,7] This paper describes the isolation, structural determination, proposed biosynthesis, and cytotoxic activities of 4 novel alkaloids (**1**–**4**) from *M. yunnanensis*.

## Results and Discussion

Compound **1** was found to possess a molecular formula of $C_{19}H_{20}N_2O$, as evidenced by high resolution electron spray ionization mass spectra (HRESIMS) at *m/z* 293.1650, in combination with $^1$H, $^{13}$C NMR, DEPT spectra, and appropriate for 11 degrees of unsaturation. The UV spectrum showed the presence of conjugated groups by

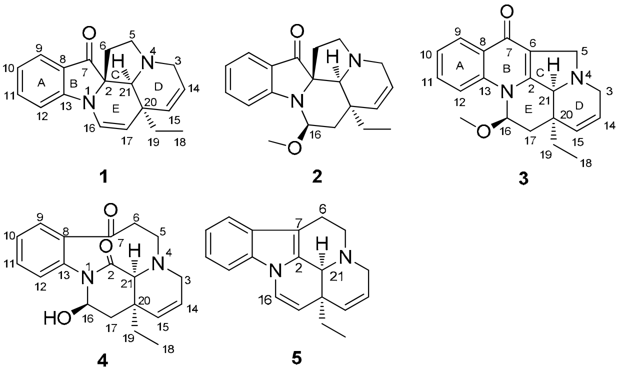

**Figure 1.** Structures of compounds **1-5**.

showing maximum absorptions at 247 and 278 nm, and the IR spectrum indicated the presence of carbonyl and olefin groups (absorption bands at 1702 and 1610 cm$^{-1}$, respectively). In the $^1$H NMR spectrum, two doublet ($\delta_H$ 7.55 and 7.13) and two triplet ($\delta_H$ 7.56 and 6.91) signals indicated that **1** was an unsubstituted indole alkaloid. In addition, signals for double bonds ($\delta_H$ 6.69 (d, $J$ = 7.0 Hz, H-16), 5.42 (dd, $J$ = 7.0, 1.4 Hz, H-17), 5.81 (m, H-14), and 5.64 (dd, $J$ = 10.0, 1.4 Hz, H-15)), a methyl group ($\delta_H$ 0.62, t, H-18), and a methylene ($\delta_H$ 0.95 and 0.87, each 1H, m, H-19) were similar to those of $\Delta^{14}$-vincamenine (**5**).[8] The $^{13}$C NMR and DEPT spectra of **1**

*To whom correspondence should be addressed. E-mail: xdluo@mail.kib.ac.cn.

showed signals of a methyl group ($\delta_C$ 8.5, q), four *sp3* methylenes ($\delta_C$ 52.1, 53.1, 39.5, and 33.0), eight *sp2* ($\delta_C$ 133.5, 126.7, 126.0, 125.0, 124.1, 120.3, 137.9, and 112.0) and one *sp3* methines ($\delta_C$ 72.1), and three *sp2* ($\delta_C$ 204.0, 159.8, and 120.8) and two *sp3* quaternary carbons ($\delta_C$ 73.9 and 42.2). In comparison with those of **5**, the four quaternary carbon signals at $\delta_C$ 131.0 (C-2), 134.8 (C-13), 129.2 (C-8), and 107.9 (C-7) of **5** were absent in **1**, instead $\delta_C$ 204.0 (s), 73.9 (s), 159.8 (s), and 120.8 (s) were present in **1**. In the heteronuclear multiple bond coherence (HMBC) spectrum of **1** (Figure 2), the correlation of $\delta_H$ 7.55 (1H, H-9) with $\delta_C$ 204.0 (s) suggested an indolone fragment, and the correlation of $\delta_H$ 6.69 (H-16), 1.88, 2.08 (H-6), and 3.10 (H-5) with $\delta_C$ 73.9 (s) led to the assignment of C-2 to a conjunct carbon of a spiral ring (Figure 1). Nuclear Overhauser Effect (NOE) correlation between H-21 and H-19 in the ROESY spectrum of **1** indicated three protons on the same side, a $\alpha$-orientation identical to the configuration of its biosynthetic precursor, $\Delta^{14}$-vincamenine.[9] Thus, the C-5 and 6 of the spiral ring (C) were on the upside of the planar indole configuration in a molecular model. Rings A, D, and E of **1** were same as those of **5**, as indicated by the HMBC and ROESY spectra.

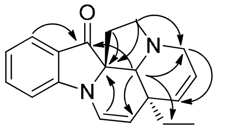

**1**

**Figure 2.** Key HMBC correlations of compound **1**.

Compound **2** possessed a molecular formula $C_{20}H_{24}N_2O_2$ according to HRESIMS (*m/z* 325.1913, $[M + H]^+$). The $^1H$ and $^{13}C$ NMR spectra were very similar to those of **1**, except for $\delta_C$ 85.5 (d), 36.2 (t), and an additional methoxyl $\delta_C$ 54.2 (q) in **2** instead of the olefinic signals at C-16/17 in **1**. This suggested an added methoxyl group at C-16 in **2**, which was further supported by the HMBC spectrum, with correlations of $\delta_H$ 5.04 (1H, t, $J$ = 8.0 Hz, H-16) with C-2, 13, and 17. In the ROESY spectrum, NOE correlation of $\delta_H$ 5.04 (H-16) with $\delta_H$ 0.77 and 0.87 (2H, m, H-19), and of $\delta_H$ 3.39 (3H, s, $OCH_3$) with 2.19 (2H, m, H-6) placed the methoxyl as $\beta$. Other structural parts of **2** were identical to **1**, as indicated by the HMBC and ROESY spectra.

Compound **3** possessed a molecular formula of $C_{20}H_{22}N_2O_2$, indicated by HRESIMS at *m/z* 323.1752 $[M + H]^+$ in combination with $^1H$, $^{13}C$ NMR, and DEPT spectra. Its UV spectrum showed a conjugation pattern (absorptions at 230 and 292 nm) different from **1**. In the $^1H$ NMR spectrum, four downfield protons ($\delta_H$ 8.34 (d, $J$ = 8.4 Hz, H-9), 7.93 (d, $J$ = 8.4 Hz, H-12), 7.68 (t, $J$ = 8.4 Hz, H-11), and 7.37 (t, $J$ = 8.4 Hz, H-10)) suggested a nonsubstituted quinolone rather than indole,[10] further supported by the HMBC correlation of $\delta_H$ 8.34 (d, H-9) with a conjugated ketone signal at $\delta_C$ 176.0 (s, C-7). Detailed comparison of the NMR data of **2** and **3**, indicated fused 6/5 rings (B and C) in **3** instead of corresponding spiral 5/5 rings in **2**. Moreover, a methoxyl in $\beta$-orientation was supported by the NOE correlation of H-16 with H-19 in its ROESY spectrum.

The HRESIMS (*m/z* 327.1708, $[M + H]^+$) of **4** established the molecular formula of $C_{19}H_{22}N_2O_3$. The NMR pattern was similar to melohenine B,[4] except for olefinic signals [$\delta_C$ 122.7 (d) and 133.4 (d) and $\delta_H$ 5.83 (1H, m) and 5.74 (1H, d, $J$ = 10.0 Hz)] observed in **4**. The double bond was assumed to be at C-14/15 from comparison of spectral data from the two compounds and was further supported by the HMBC spectrum of **4**, with correlations of $\delta_C$ 39.6 (s, C-20) with $\delta_H$ 5.83 (1H, m, H-14) and 5.74 (1H, d, H-15). NOE correlations of H-21 with H-19 and H-16 supported a 16$\beta$-OH for **4**, identical to melohenine B.

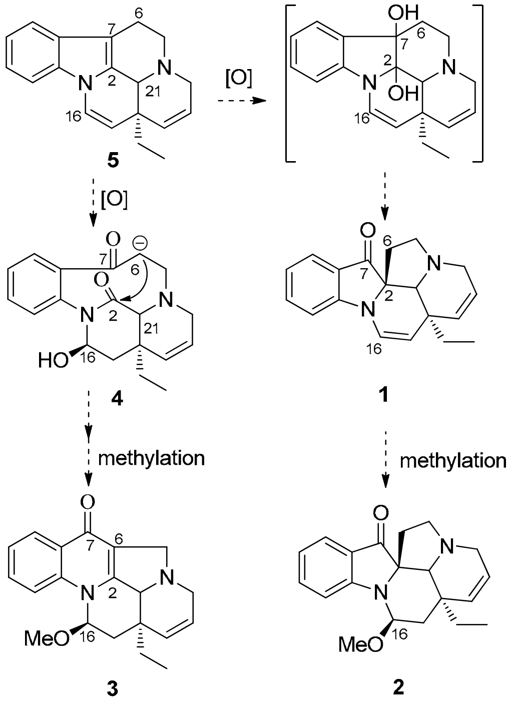

**Figure 3.** Proposed biogenetic relationships of **1**-**5**.

The possible biosynthetic relationships of these new compounds in which they were derived from a common precursor **5** was proposed here (Figure 3). Different oxidation processes may have produced two kinds of intermediates, from which **4** was isolated. Further rearrangement then formed two new skeletons, including the spiro-indolone alkaloids (**1** and **2**), and a quinolone alkaloid (**3**). To our knowledge, this is the first report of the co-occurrence of monoterpenoid indoles (**1**, **2**, and **5**), a quinoline (**3**), and their key intermediates (**4**) in same plant, supporting the biosynthesis of quinoline from indole and concurring with previous *in vivo* tracer experiments in the literature.

Compounds **1**–**5** were evaluated for their cytotoxicity against five human cancer cell lines, MCF-7 breast, SMMC-7721 hepatocellular carcinoma, HL-60 myeloid leukemia, SW480 colon cancer, and A-549 lung cancer. Compound **2** showed cytotoxicity against all cell lines, with $IC_{50}$ values of 14.24, 19.08, 15.48, 13.29, and 40.0 $\mu$M, respectively, while the cisplatin control showed $IC_{50}$ values of 21.90, 15.24, 1.05, 19.92, and 9.40 $\mu$M. The other compounds were inactive ($IC_{50}$ > 40 $\mu$M).

**Experimental Section**

**Table 1. $^{1}$H and $^{13}$C NMR data for meloyunines A-C (1-3) and 14,15-didehydromelohenine B (4). (*J* in Hz, $\delta$ in ppm).**

| Pos. | meloyunine A (**1**)[c] | | meloyunine B (**2**)[c] | | meloyunine C (**3**)[d] | | 14,15-didehydromelohenine B (**4**)[d] | |
|---|---|---|---|---|---|---|---|---|
| | $\delta_H$[a] | $\delta_C$[b] | $\delta_H$[a] | $\delta_C$[b] | $\delta_H$[b] | $\delta_C$[b] | $\delta_H$[b] | $\delta_C$[b] |
| 2 | | 73.9 s | | 73.4 s | | 153.7 t | | 170.6 s |
| 3 | 3.41 (1H, m) | 52.1 t | 3.41 (1H, m) | 52.1 t | 3.14 (1H, dd, 15.0, 4.8) | 47.6 t | 3.18 (2H, m,) | 53.3 t |
| | 2.84 (1H, d, 16.0) | | 2.88 (1H, d, 12.0) | | 2.70 (dd, 15.0, 2.5) | | | |
| 5 | 3.10 (1H, m) | 53.1 t | 3.20 (1H, m) | 52.6 t | 3.68 (1H, d, 14.0) | 57.5 t | 3.42 (1H, m) | 53.3 t |
| | 2.31 (1H, m) | | 2.35 (1H, m) | | 3.93 (1H, brd, 14.0) | | 3.24 (1H, m) | |
| 6 | 1.87 (1H, m) | 39.5 t | 1.97 (1H, m) | 37.7 t | | 117.5 s | 3.60 (1H, dd, 7.8, 13.0) | 42.2 t |
| | 2.08 (1H, m) | | 2.19 (1H, m) | | | | 2.60 (1H, dd, 13.0, 10.0) | |
| 7 | | 204.0 s | | 205.7 s | | 176.0 s | | 202.9 s |
| 8 | | 120.8 s | | 119.5 s | | 128.0 s | | 138.8 s |
| 9 | 7.55 (1H, d, 7.5) | 125.0 d | 7.52 (1H, d, 8.0) | 125.0 d | 8.34 (1H, d, 8.4) | 127.0 d | 7.48 (1H, d, 7.5) | 128.5 d |
| 10 | 6.91 (1H, t, 7.5) | 120.3 d | 6.88 (1H, t, 8.0) | 119.8 d | 7.37 (1H, t, 8.4) | 124.0 d | 7.32 (1H, t, 7.5) | 126.9 d |
| 11 | 7.56 (1H, t, 7.5) | 137.9 d | 7.57 (1H, t, 8.0) | 138.3 d | 7.68 (1H, t, 8.4) | 131.9 d | 7.55 (1H, t, 7.5) | 132.1 d |
| 12 | 7.13 (1H, d, 7.5) | 112.0 d | 7.24 (1H, d, 8.0) | 110.6 d | 7.93 (1H, d, 8.4) | 116.1 d | 7.24 (1H, d, 7.5) | 123.5 d |
| 13 | | 159.8 s | | 162.5 s | | 141.7 s | | 138.4 s |
| 14 | 5.81 (1H, m) | 126.7 d | 5.72 (1H, m) | 126.6 d | 5.74 (1H, m) | 125.1 d | 5.83 (1H, m) | 122.7 d |
| 15 | 5.64 (1H, dd, 10.0, 1.4) | 133.5 d | 5.46 (1H, d, 10.0) | 133.5 d | 5.60 (1H, br.d, 10.0) | 132.2 d | 5.74 (1H, d, 10.0) | 133.4 d |
| 16 | 6.69 (1H, d, 7.0) | 124.1 d | 5.04 (1H, t, 8.0) | 85.5 d | 6.10 (1H, t, 3.2) | 84.3 d | 5.81 (1H, d, 3.6,) | 84.6 d |
| 17 | 5.42 (1H, dd, 7.0, 1.4) | 126.0 d | 2.38 (1H, m) | 36.2 t | 2.46 (1H, dd, 15.0, 4.0) | 40.8 t | 2.43 (1H, d, 14.0) | 42.9 d |
| | | | 2.08 (1H, m) | | 1.80 (1H, dd, 15.0, 3.0) | | 2.07 (1H, dd, 3.6,14.0) | |
| 18 | 0.62 (3H, t, 7.5) | 8.5 q | 0.60 (1H, t, 7.5) | 8.0 q | 0.98 (3H, t, 7.5) | 9.6 q | 0.89 (3H, t, 7.5) | 7.9 q |
| 19 | 0.95 (1H, m) | 33.0 t | 0.87 (1H, m) | 27.5 t | 2.12 (1H, m) | 37.2 t | 1.49 (2H, m) | 34.5 t |
| | 0.87 (1H, m) | | 0.77 (1H, m) | | 1.60 (1H, m) | | | |
| 20 | | 41.2 s | | 39.9 s | | 35.9 s | | 39.6 s |
| 21 | 2.77 (1H, s) | 72.1 d | 2.34 (1H, s) | 67.7 d | 4.21 (1H, s) | 65.9 d | 3.18 (1H, s) | 71.8 d |
| OMe | | | 3.39 (3H, s) | 54.2 q | 3.39 (3H, s) | 55.8 q | | |

[a]Data recorded on a Bruker avance-600 MHz spectrometer, [b]in Bruker AV-400 MHz, [c]in acetone-$d_6$, [d]in $CDCl_3$

**General Experimental Procedures.** Optical rotations were measured with a Jasco P-1020 spectropolarimeter. UV spectra were recorded on a Shimadzu double-beam 210A spectrophotometer. IR (KBr) spectra were obtained on Bruker Tensor 27 infrared spectrophotometer. $^{1}$H, $^{13}$C and 2D NMR spectra were recorded on a Bruker avance III-600 and AV-400 MHz NMR spectrometer with TMS as internal standard. MS data were obtained on API Qstar Pulsar I spectrometer. C18 silica gel (20–45 $\mu$m) was bought from Fuji Chemical Ltd., Japan. MPLC was employed Büchi pumps system coupled with glass column (15 × 230 and 26 × 460 mm, respectively, C18 silica gel). HPLC was performed using Waters 600 pumps coupled with analytical and semipreparative Sunfire C18 columns (4.6 × 150 and 19 × 150 mm, respectively). The HPLC system employed a Waters 2996 photodiode array detector and a Waters fraction collector II.

**Plant Materials.** Leaves and twigs of *M. yunnanensis* collected in Apr. 2009 in Honghe of Yunnan Province, P. R. China, and identified by Dr. De-Shan Deng. Voucher specimen (Cai091106) was deposited in the State Key Laboratory of Phytochemistry and Plant Resources in West China, Kunming Institute of Botany, Chinese Academy of Sciences.

**Extraction and Isolation.** Dried and powdered leaves and twigs of *M. yunnanensis* (40 kg) were extracted three times with methanol (MeOH) at room temperature and the solvent evaporated *in vacuo*. The residue was dissolved in 0.3% aqueous hydrochloric acid, and the solution subsequently basified using ammonia water to pH 9–10. The basic solution was partitioned with EtOAc, producing an aqueous and EtOAc phase. The resulting EtOAc fraction (205 g) was collected and then subjected to column chromatography over silica gel and eluted with a chloroform-acetone gradient (1/0 to 3/1, v/v) to afford five fractions (I–VII), which were concentrated *in vacuo*. Fraction II (5 g) was further chromatographed using a petroleum ether-acetone gradient (19/1 to 9/1, v/v) as the eluent to yield 8 subfractions, II-1–II-8. Compound **4** (46 mg) was crystallized from II-8 (90 mg). Subfraction II-1 (112 mg) was separated by semi-preparative reversed-phase $C_{18}$-HPLC on a Sunfire column (19 × 250 mm) with a gradient flow of 70–75% aqueous MeOH to yield **2** (5 mg). II-3 (120 mg) was further purified on a same semi-preparative column with a gradient flow of 65–80% aqueous MeOH to afford **5** (107 mg). Similar semi-preparative column separations with gradient flows of 60–75% aqueous MeOH were used to fractionate II-4 (90 mg) and II-6 (100 mg) to produce **1** (4 mg) and **3** (5 mg), respectively.

**Meloyunine A** (**1**): white powder; $[\alpha]^{20}_{D}$ + 223 (*c* 0.16, MeOH); UV (MeOH) $\lambda_{max}$ (log ε) 247 (3.37), 278 (3.21) nm; IR (KBr) $\nu_{max}$ 3431, 1702, 1610 $cm^{-1}$; $^1H$ and $^{13}C$ NMR data, Table 1; ESIMS *m/z* 293 $[M + H]^+$; positive ion HRESIMS *m/z* 293.1650 (calcd for $C_{19}H_{21}N_2O$ $[M + H]^+$, 293.1653).

**Meloyunine B** (**2**): white powder; $[\alpha]^{20}_{D}$ + 181 (*c* 0.15, MeOH); UV (MeOH) $\lambda_{max}$ (log ε) 246 (3.37), 280 (2.80) nm; IR (KBr) $\nu_{max}$ 3433, 1703, 1613 $cm^{-1}$; $^1H$ and $^{13}C$ NMR data, Table 1; ESIMS *m/z* 325 $[M + H]^+$; positive ion HRESIMS *m/z* 325.1913 (calcd for $C_{20}H_{25}N_2O_2$ $[M + H]^+$, 325.1916).

**Meloyunine C** (**3**): white powder; $[\alpha]^{20}_{D}$ + 141 (*c* 0.13, MeOH); UV (MeOH) (log ε) $\lambda_{max}$ 230 (3.46), 292 (3.15) nm; IR (KBr) $\nu_{max}$ 3431, 1708, 1623, 1592 $cm^{-1}$; $^1H$ and $^{13}C$ NMR data, Table 1; ESIMS *m/z* 323 $[M + H]^+$; positive ion HRESIMS *m/z* 323.1752 (calcd for $C_{20}H_{23}N_2O_2$ $[M + H]^+$, 323.1759).

**14,15-Didehydromelohenine B** (**4**): white powder; $[\alpha]^{20}_{D}$ + 133 (*c* 0.06, MeOH); UV (MeOH) (log ε) $\lambda_{max}$ 228 (3.35), 298 (3.14) nm; IR (KBr) $\nu_{max}$ 3483, 1686, 1668, 1598 $cm^{-1}$; $^1H$ and $^{13}C$ NMR data, Table 1; ESIMS *m/z* 327 $[M + H]^+$; positive ion HRESIMS *m/z* 327.1708 (calcd for $C_{19}H_{23}N_2O_3$ $[M + H]^+$, 327. 1708).

**Cytotoxicity Assay.** Five human cancer cell lines, MCF-7 breast, SMMC-7721 hepatocellular carcinoma, HL-60 myeloid leukemia, SW480 colon cancer, and A-549 lung cancer, were used in the cytotoxic assay. Cells were cultured in RPMI-1640 or in DMEM medium (Hyclone, USA), supplemented with 10% fetal bovine serum (Hyclone, USA) in 5% $CO_2$ at 37 °C. The cytotoxicity assay was performed according to the MTT (3-(4,5-dimethylthiazol-2-yl)-2,5-diphenyl tetrazolium bromide) method in 96-well microplates.[11] Briefly, 100 $\mu$L of adherent cells was seeded into each well of 96-well cell culture plates and allowed to adhere for 12 h before addition of test compounds, while suspended cells were seeded just before drug addition with initial density of $1 \times 10^5$ cells/mL. Each tumor cell line was exposed to the test compound at concentrations of 0.0625, 0.32, 1.6, 8, and 40 $\mu$M in triplicates for 48 h, with cisplatin (Sigma, USA) as positive control. After compound treatment, cell viability was detected and a cell growth curve was graphed. $IC_{50}$ values were calculated by Reed and Muench's method.[12]

## Electronic Supplementary Material

Supplementary material is available in the online version of this article at http://dx.doi.org/10.1007/s13659-011-0001-0 and is accessible for authorized users.

## Acknowledgments

This work was supported in part by the National Natural Science Foundation of China (2107298), the 973 Program of Ministry of Science and Technology of P. R. China (2009CB522300), and Chinese Academy of Sciences (KSCX2-EW-R-15 and XiBuZhiGuang Project).

**Open Access** This article is distributed under the terms of the Creative Commons Attribution License which permits any use, distribution, and reproduction in any medium, provided the original author(s) and source are credited.

## Electronic supplementary material

Supplementary material, approximately 2.56 MB.
