# Supplementary material for: Novel indole and quinoline alkaloids from Melodinus yunnanensis
Source: Nat Prod Bioprospect. 2011 Sep 2;1(1):25–8. doi: 10.1007/s13659-011-0001-0 (PMC4131712; doi:10.1007/s13659-011-0001-0)

Electronic Supplementary Material

Novel indole and quinoline alkaloids from *Melodinus yunnanensis*

Xiang-Hai CAI, Yan LI, Jia SU, Ya-Ping LIU, Xiao-Ning LI, Xiao-Dong LUO*

State Key Laboratory of Phytochemistry and Plant Resources in West China, Kunming Institute of Botany, Chinese Academy of Sciences, Kunming 650201, China

Received 8 June 2011; Accepted 4 July 2011

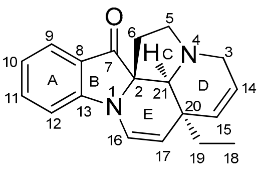

1

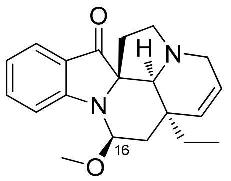

2

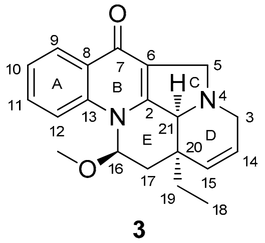

3

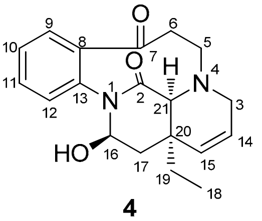

4

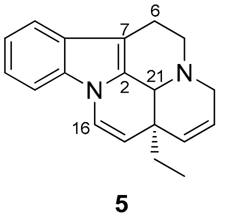

5

Structures of compounds **1**–**5**.

*To whom correspondence should be addressed. E-mail: xdluo@mail.kib.ac.cn.

Table of Contents

Figure 1. ^{1}H NMR spectrum of meloyunine A. (**1**)

Figure 2. ^{13}C NMR spectrum of meloyunine A (**1**)

Figure 3. HSQC spectrum of meloyunine A. (**1**)

Figure 4. HMBC spectrum of meloyunine A (**1**)

Figure 5. ROESY spectrum of meloyunine A (**1**)

Figure 6. ^{1}H NMR spectrum of meloyunine B (**2**)

Figure 7. ^{13}C NMR spectrum of meloyunine B (**2**)

Figure 8. HSQC spectrum of meloyunine B. (**2**)

Figure 9. HMBC spectrum of meloyunine B (**2**)

Figure 10. ROESY spectrum of meloyunine B (**2**)

Figure 11. ^{1}H NMR spectrum of meloyunine C (**3**)

Figure 12. ^{13}C NMR spectrum of meloyunine C (**3**)

Figure 13. HSQC spectrum of meloyunine C (**3**)

Figure 14. HMBC spectrum of meloyunine C (**3**)

Figure 15. ROESY spectrum of meloyunine C (**3**)

Figure 16. ^{1}H NMR spectrum of 14,15-dehydromelohenine B (**4**)

Figure 17. ^{13}C NMR spectrum of 14,15-dehydromelohenine B (**4**)

Figure 18. HSQC spectrum of 14,15-dehydromelohenine B (**4**)

Figure 19. HMBC spectrum of 14,15-dehydromelohenine B (**4**)

Figure 20. ROESY spectrum of 14,15-dehydromelohenine B (**4**)

Figure 21. ^{1}H NMR spectrum of Δ^{14}-vincamenine (**5**)

Figure 1. ^{1}H NMR spectrum of meloyunine A. (**1**)

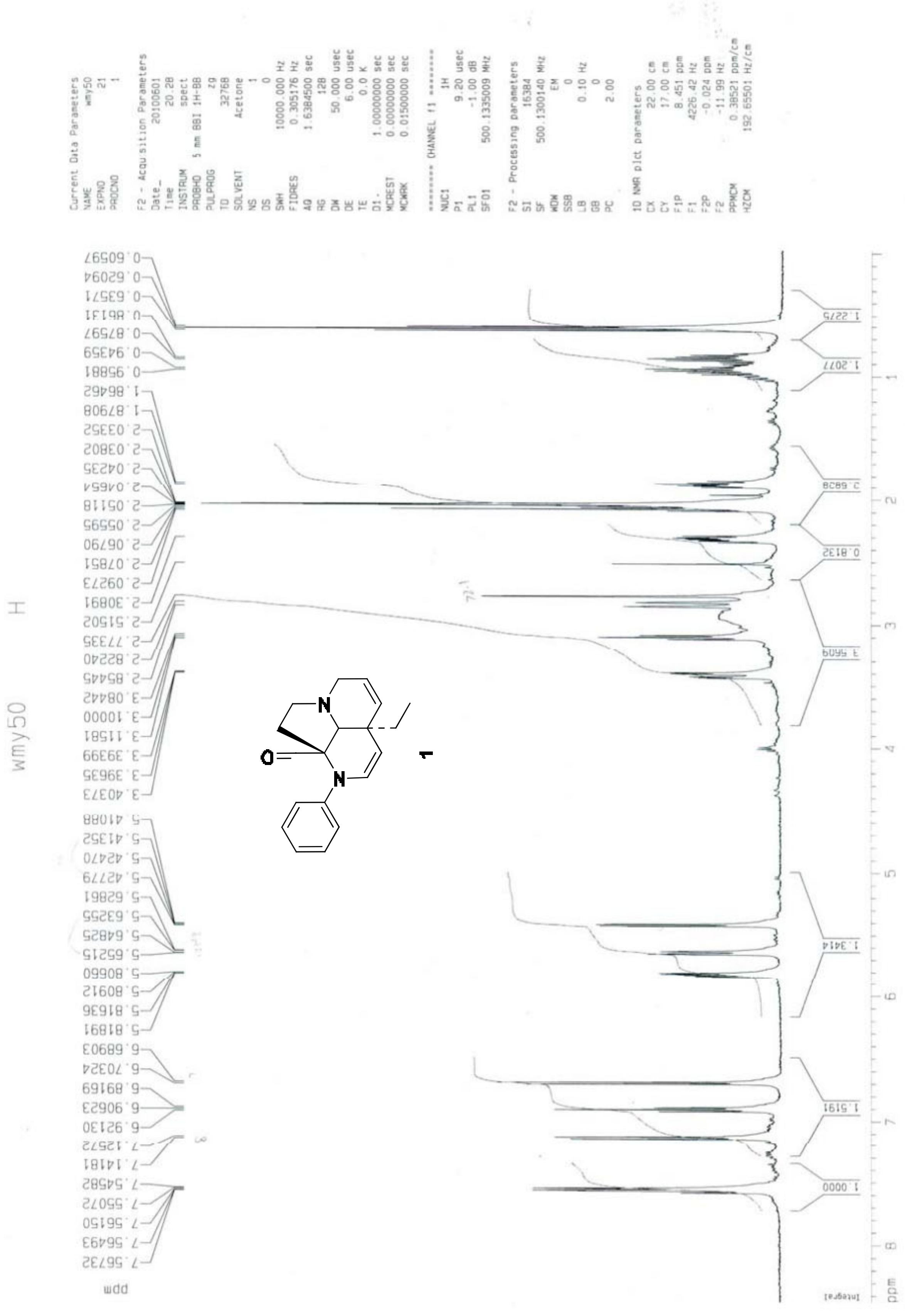

Figure 2. ^{13}C NMR spectrum of meloyunine A (**1**)

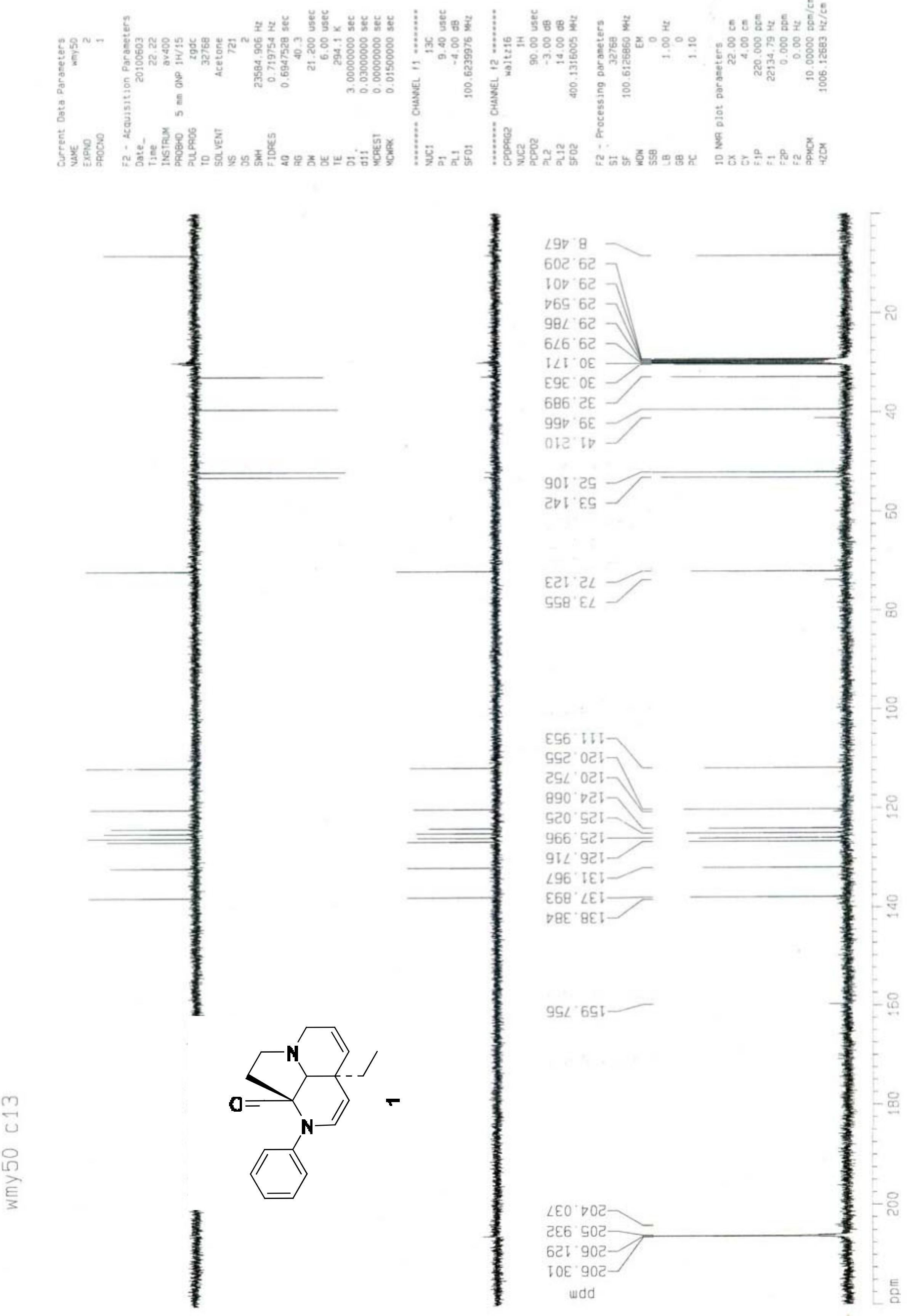

Figure 3. HSQC spectrum of meloyunine A. (**1**)

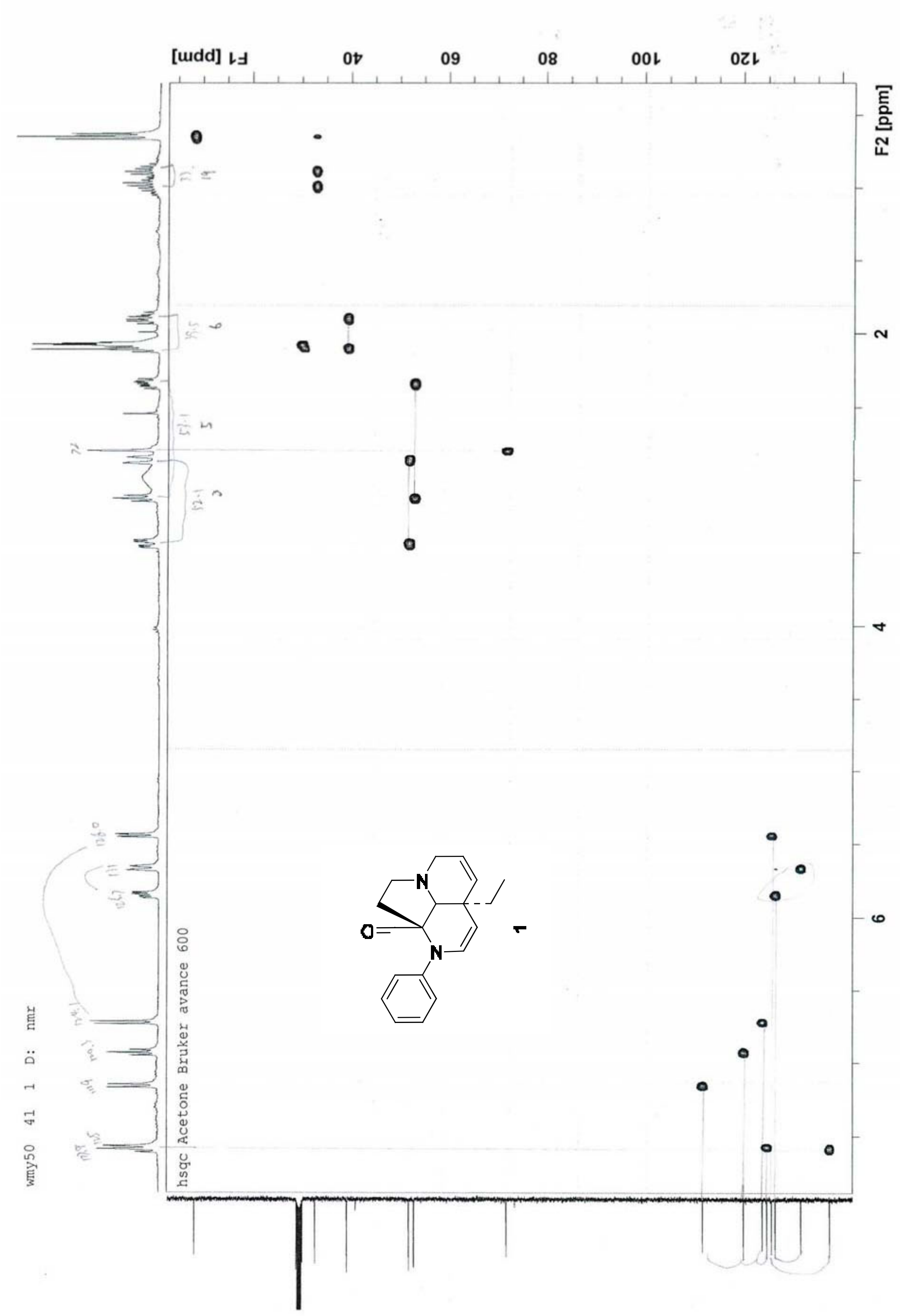

Figure 4. HMBC spectrum of meloyunine A (**1**)

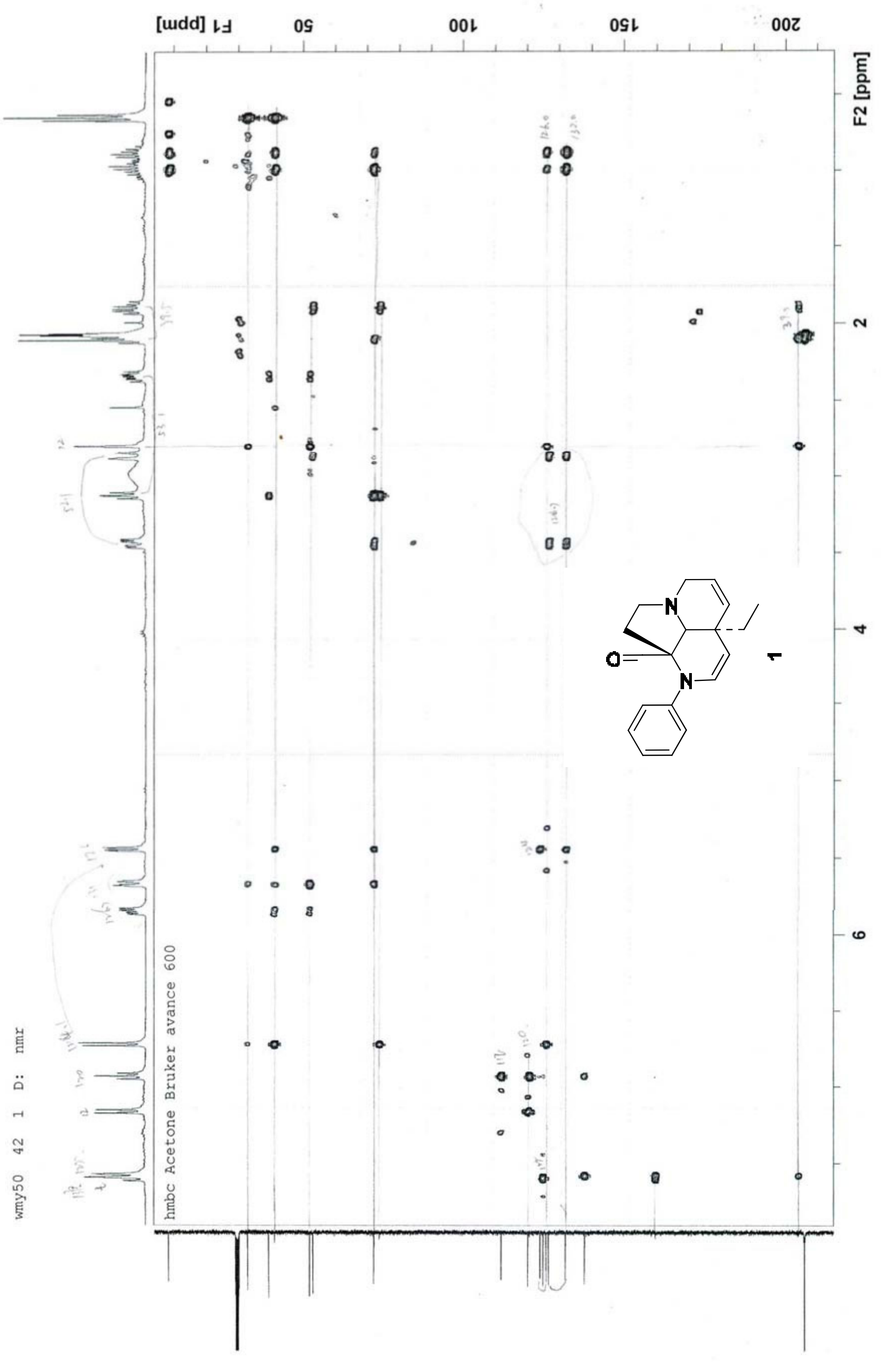

Figure 5. ROESY spectrum of meloyunine A (**1**)

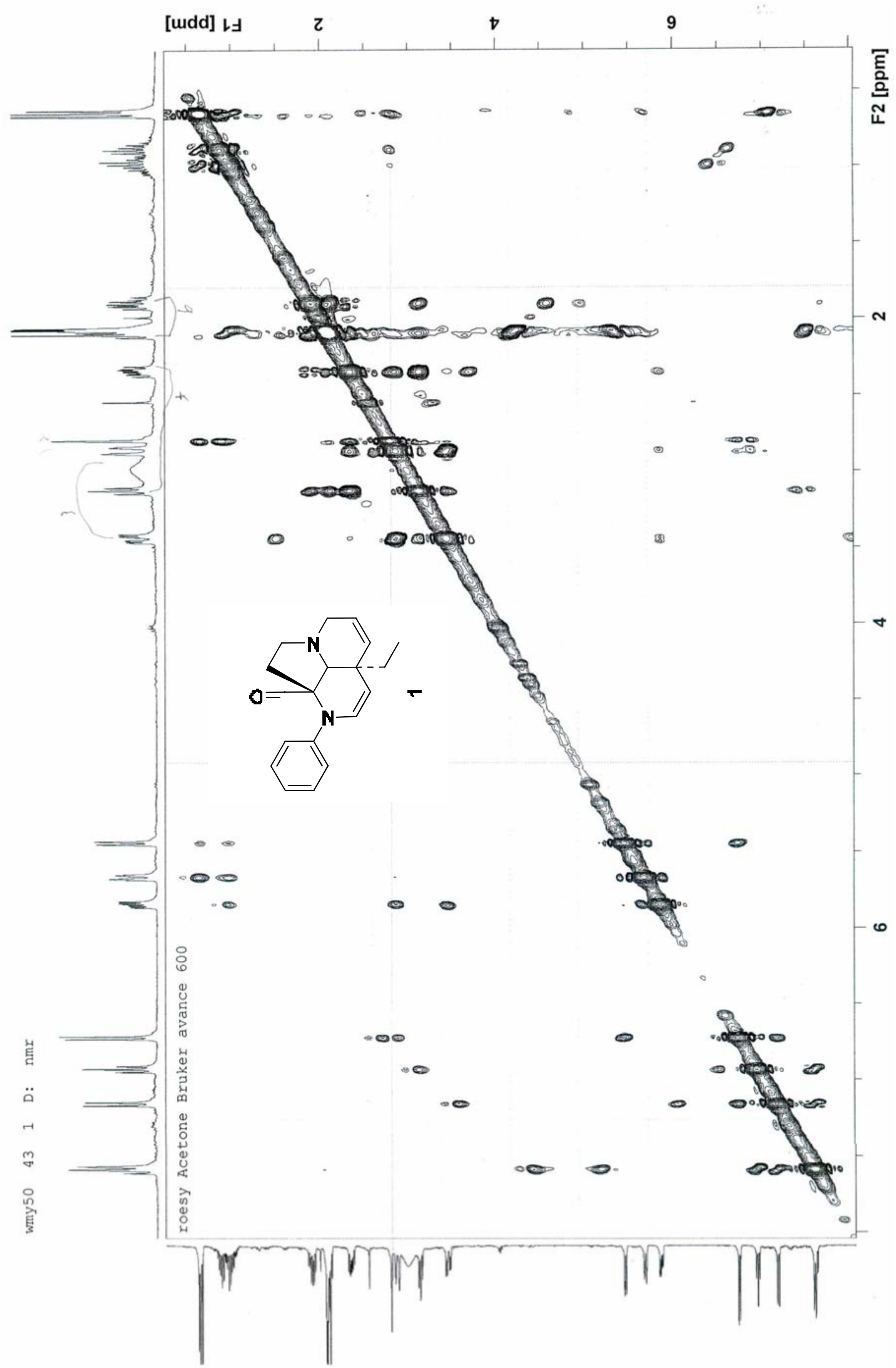

Figure 6. ^{1}H NMR spectrum of meloyunine B (**2**)

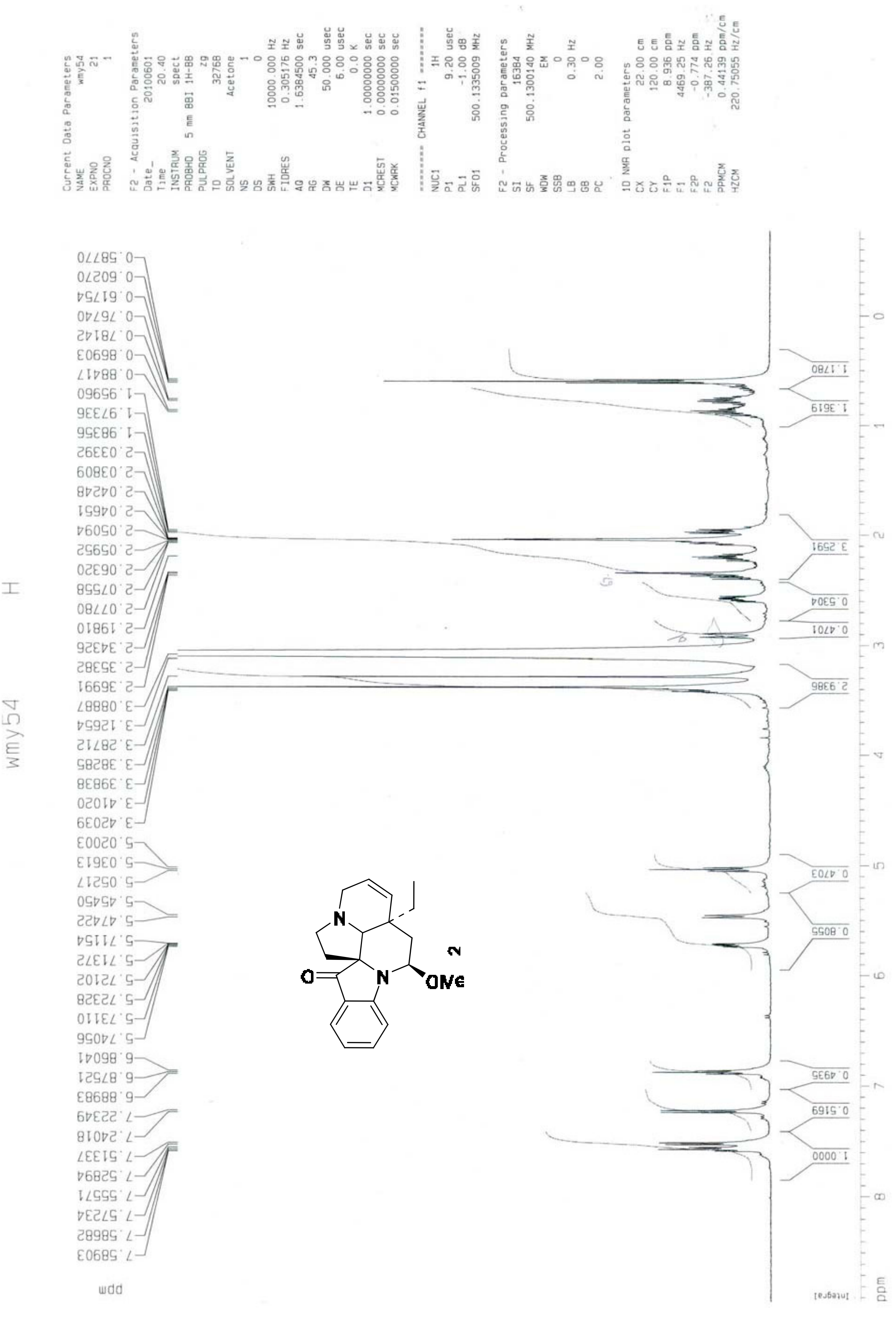

Figure 7. ^{13}C NMR spectrum of meloyunine B (**2**)

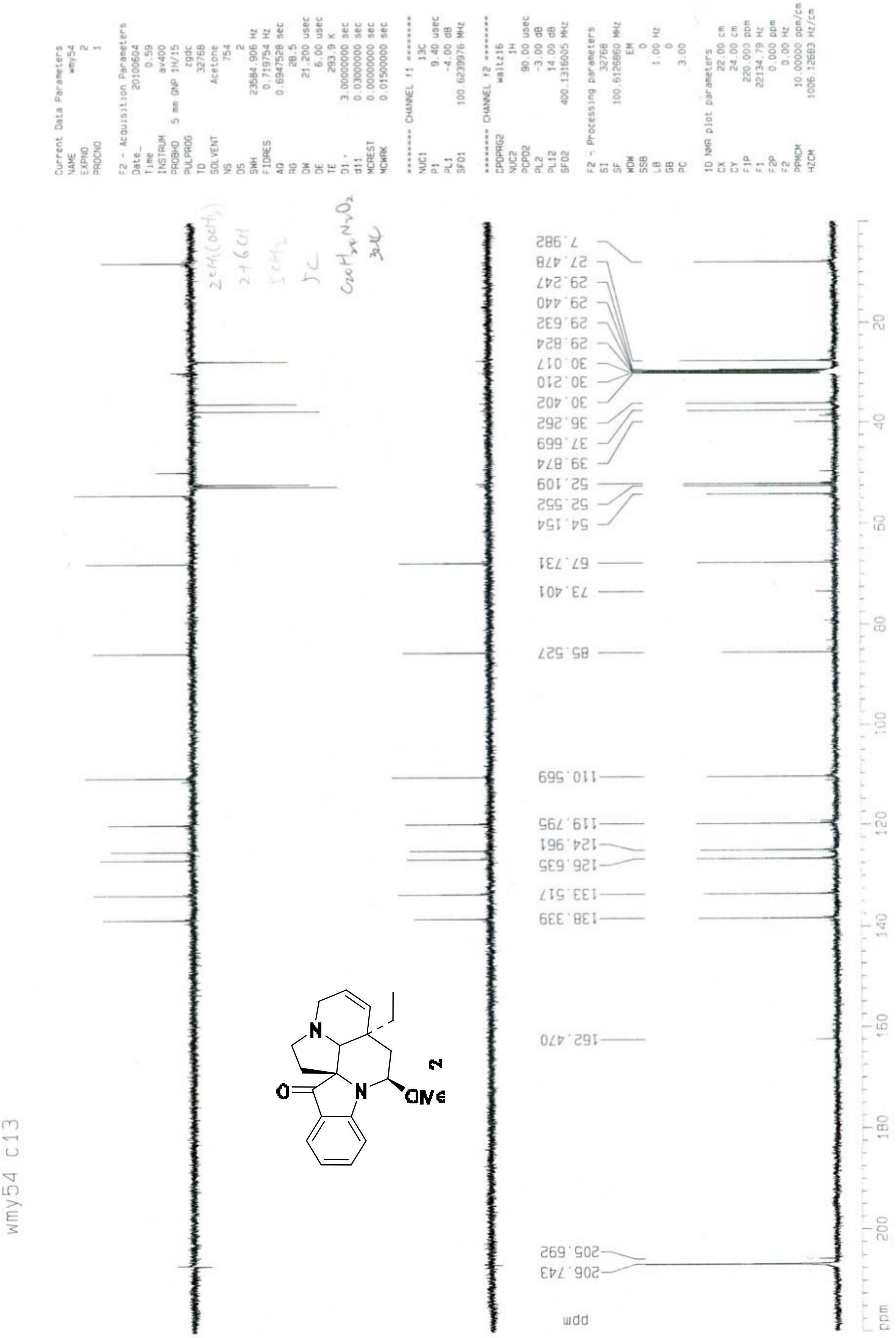

Figure 8. HSQC spectrum of meloyunine B. (**2**)

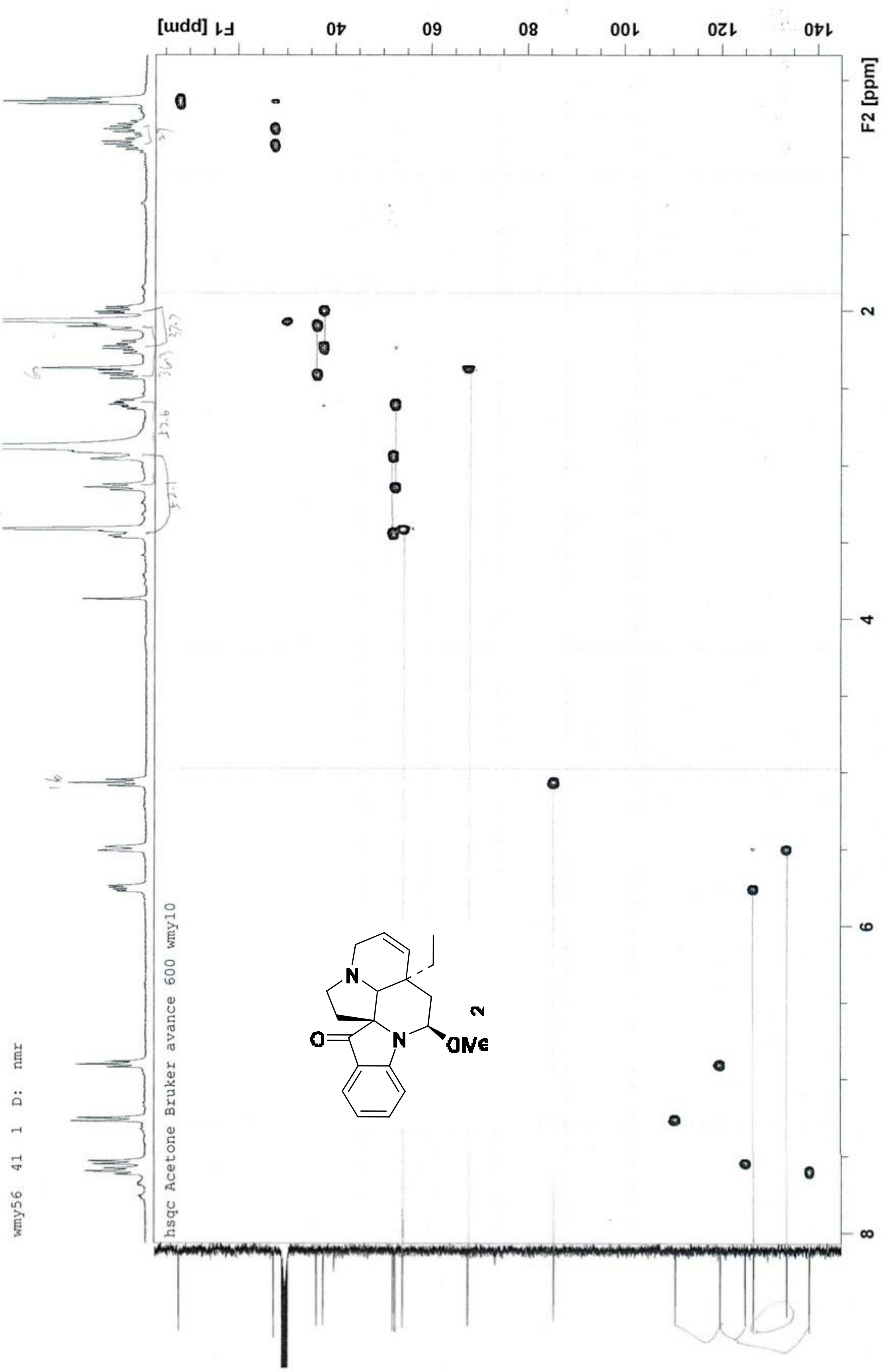

Figure 9. HMBC spectrum of meloyunine B (**2**)

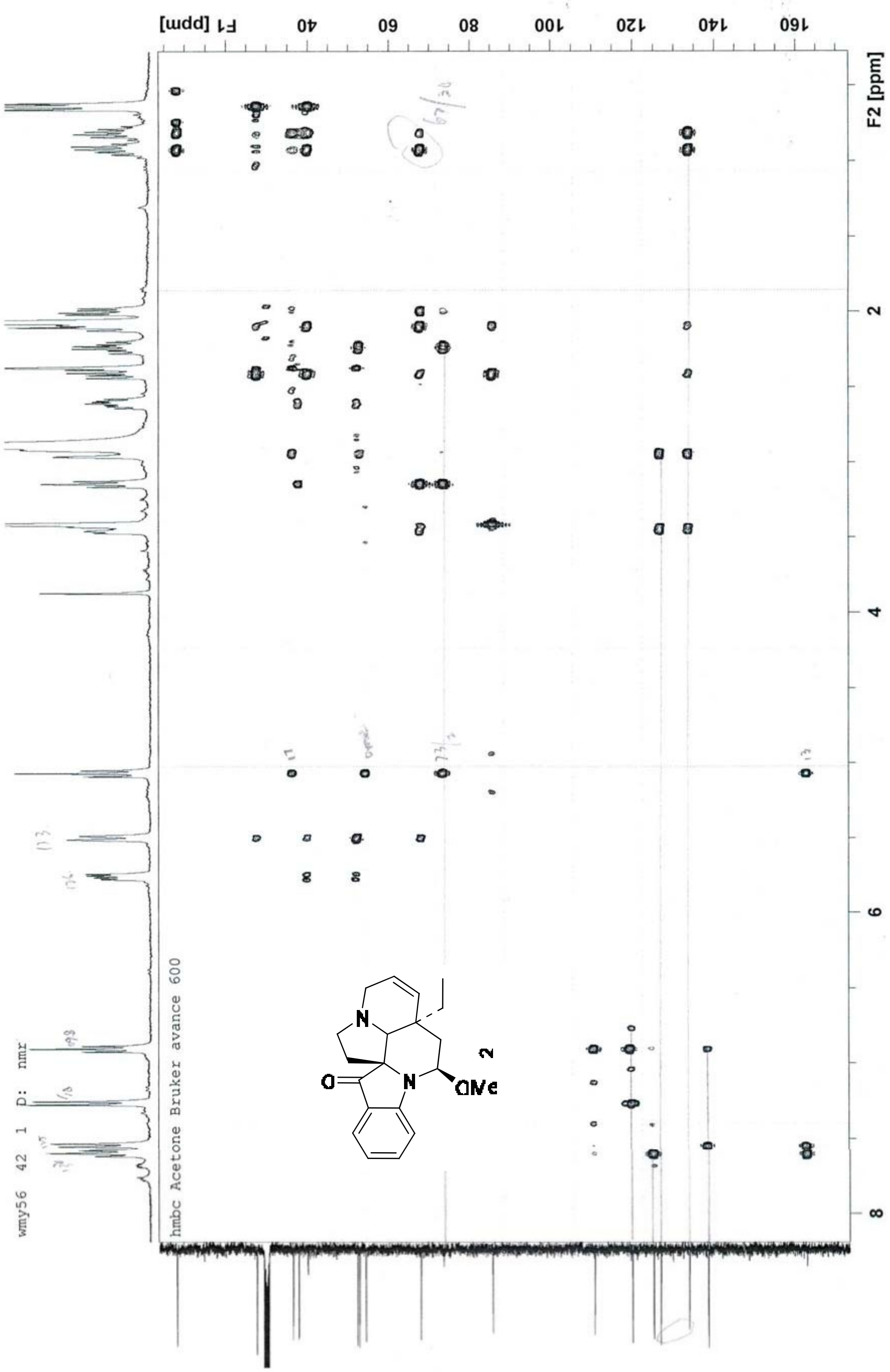

Figure 10. ROESY spectrum of meloyunine B (**2**)

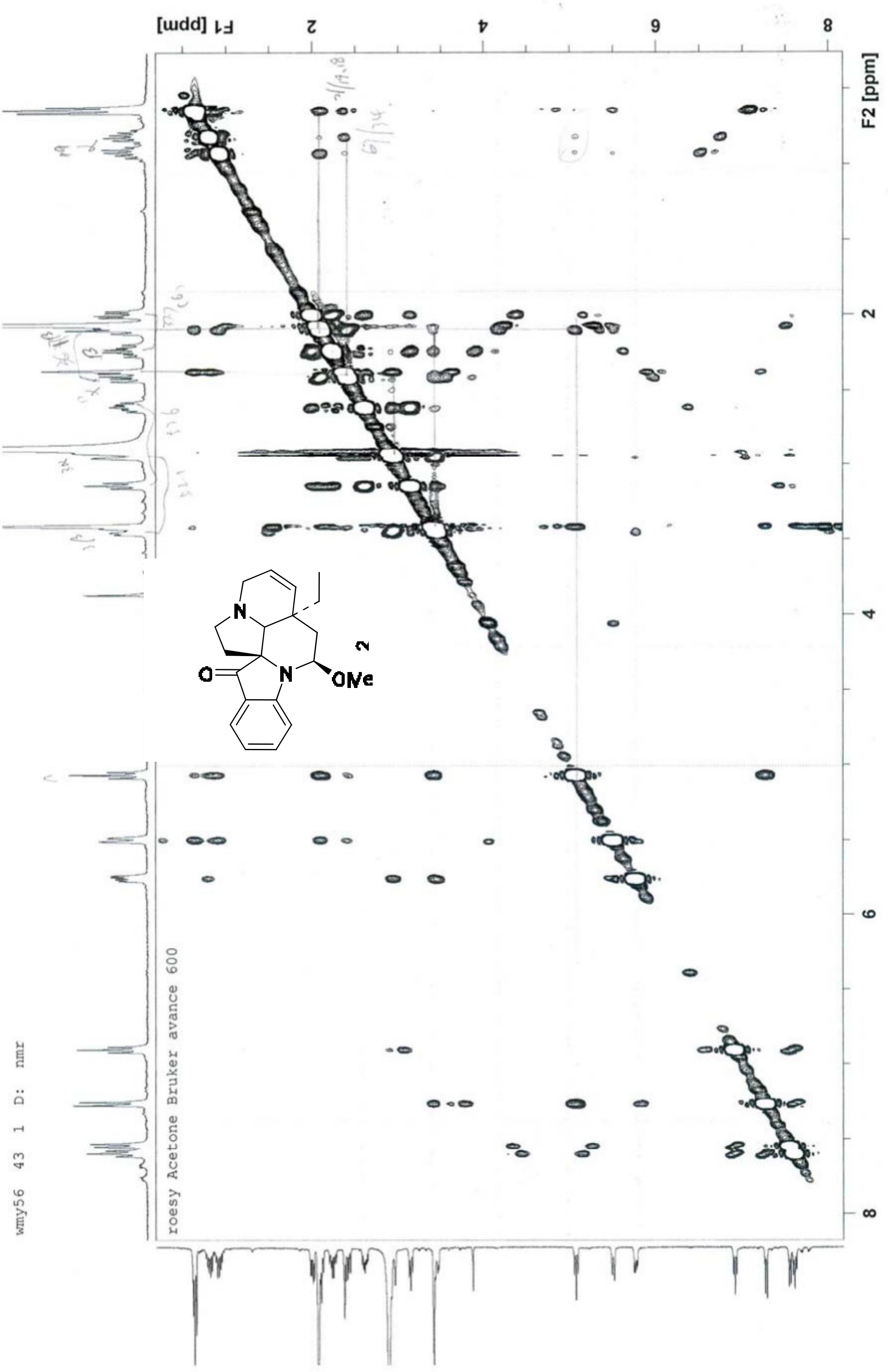

Figure 11. ^{1}H NMR spectrum of meloyunine C (**3**)

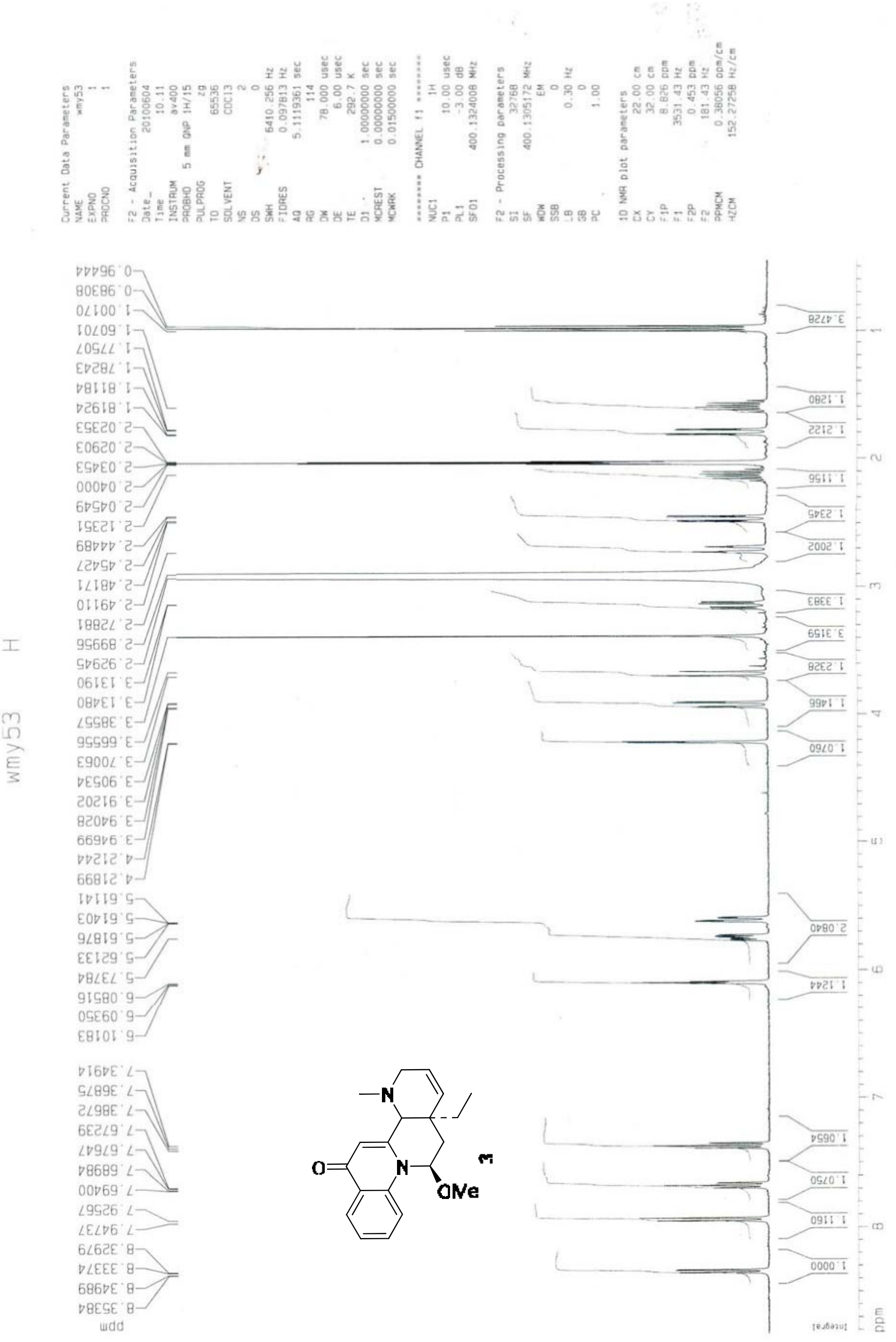

Figure 12. ^{13}C NMR spectrum of meloyunine C (**3**)

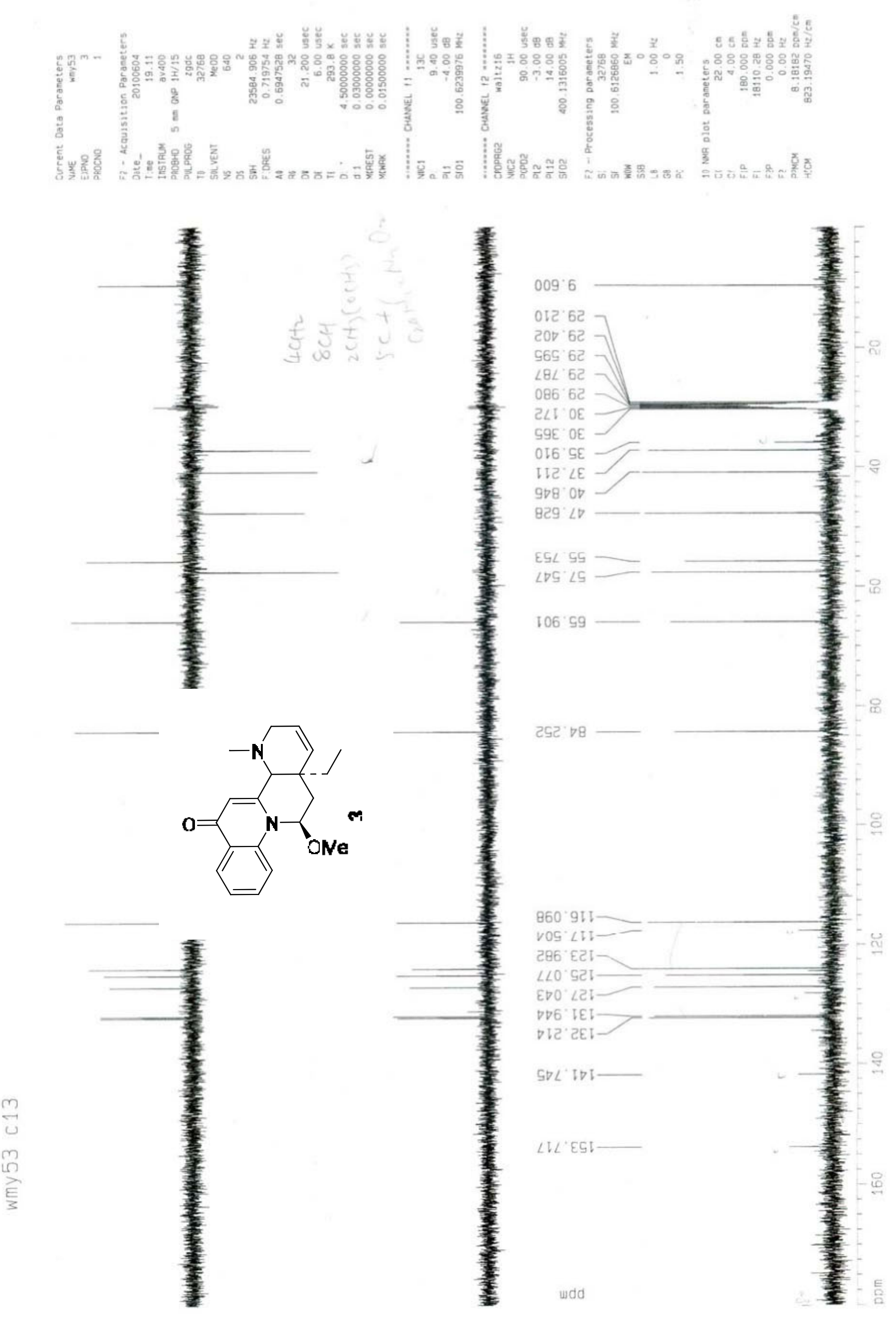

Figure 13. HSQC spectrum of meloyunine C (**3**)

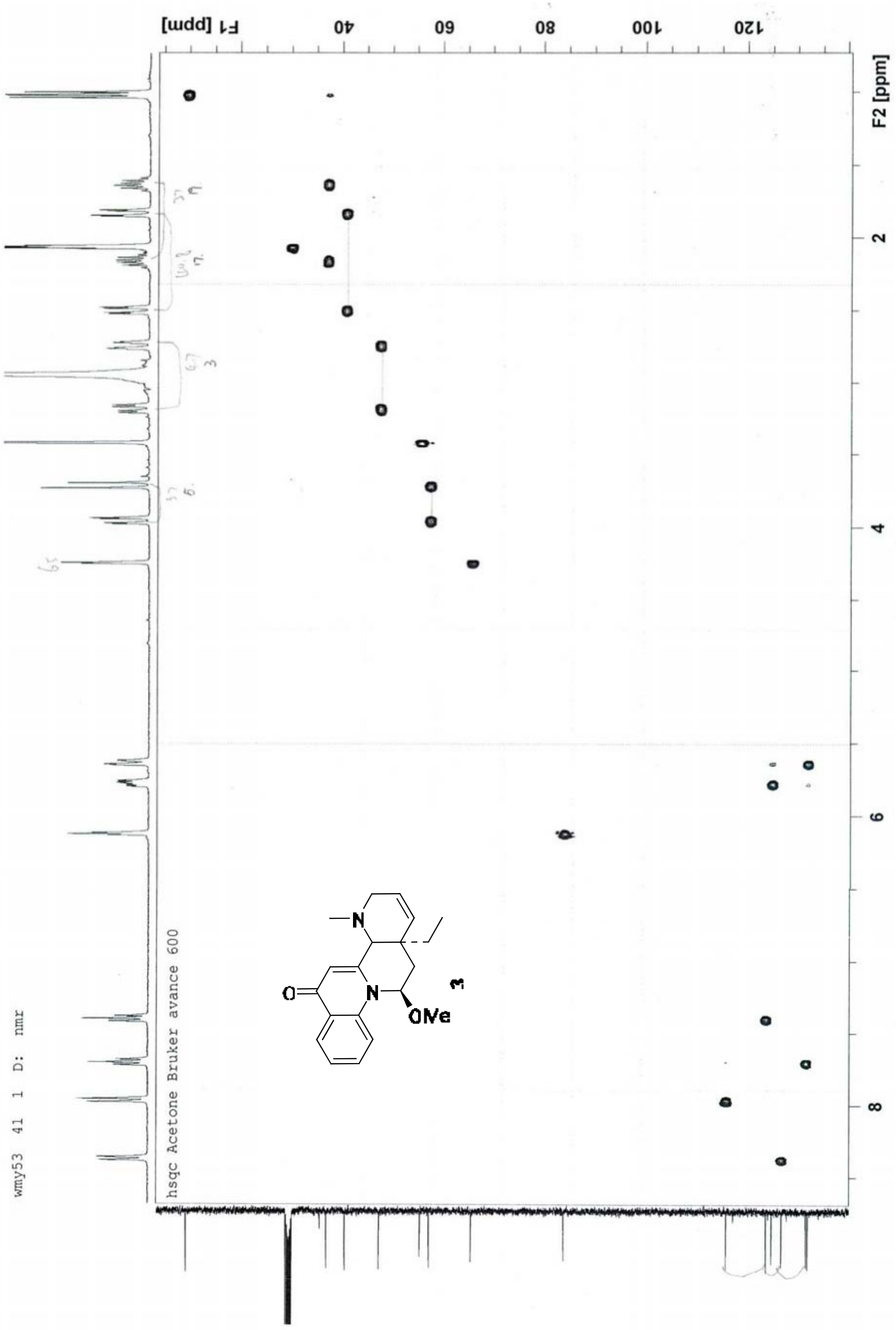

Figure 14. HMBC spectrum of meloyunine C (**3**)

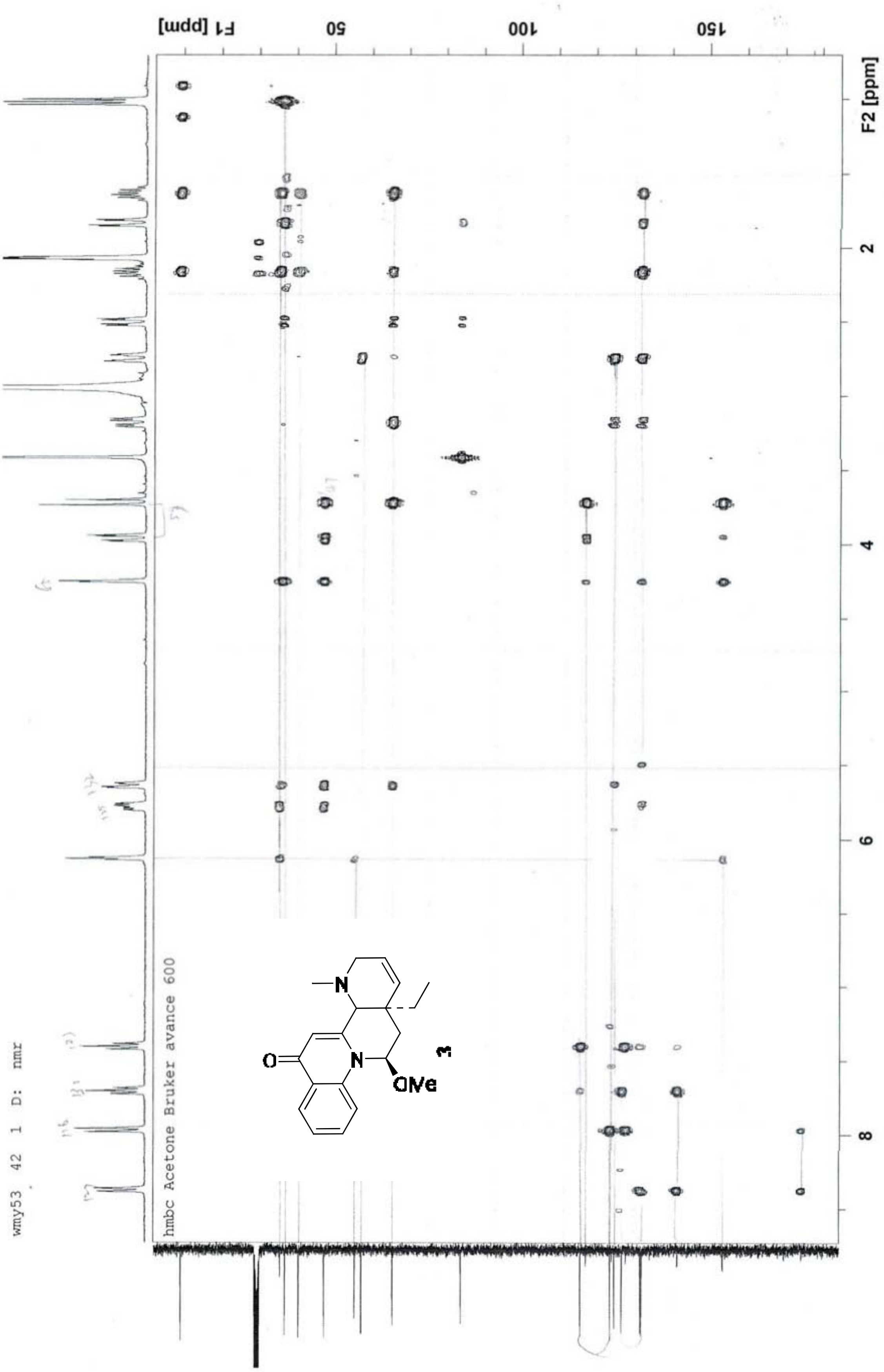

Figure 15. ROESY spectrum of meloyunine C (**3**)

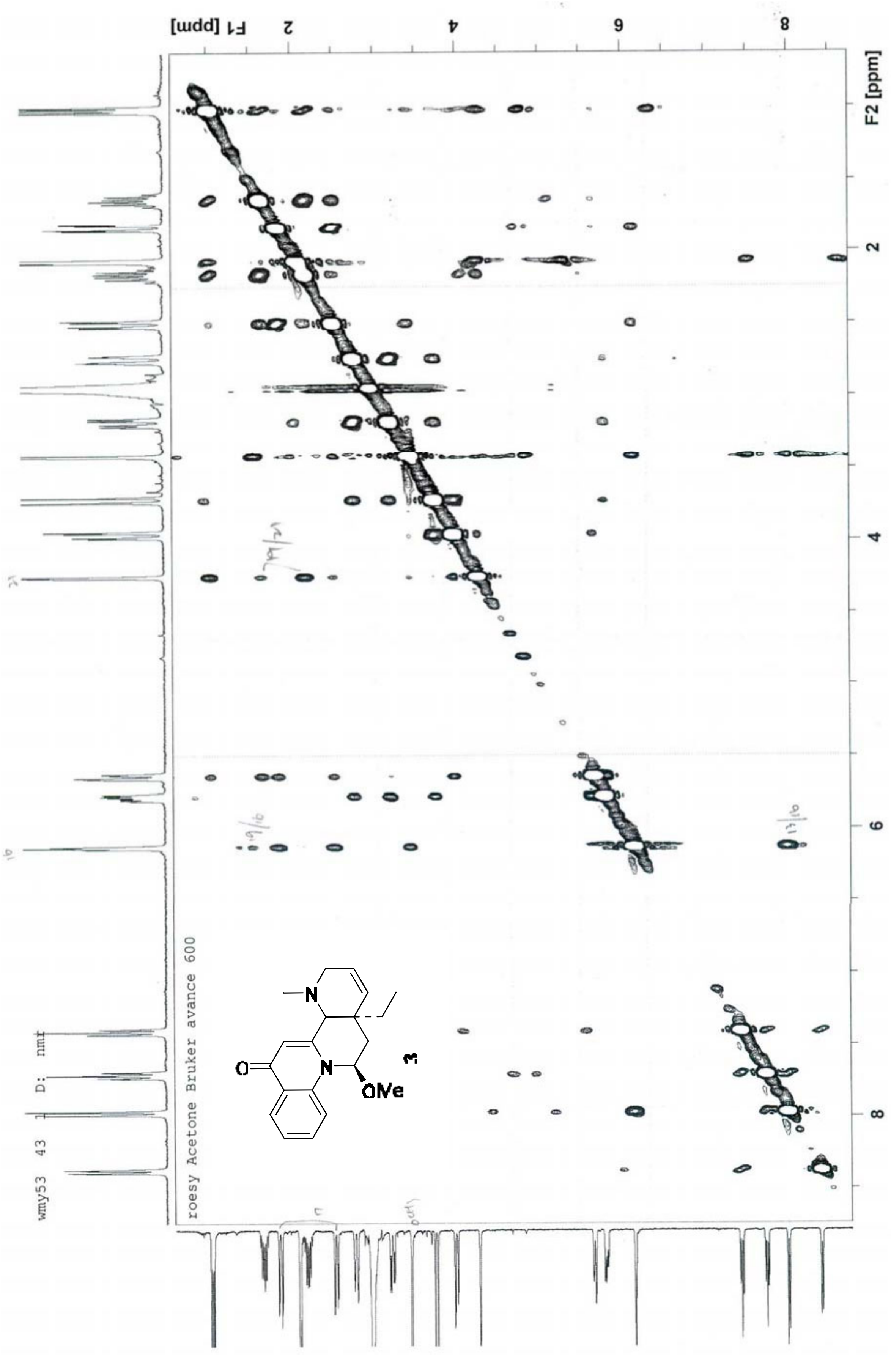

Figure 16. ^{1}H NMR spectrum of 14,15-dehydromelohenine B (**4**)

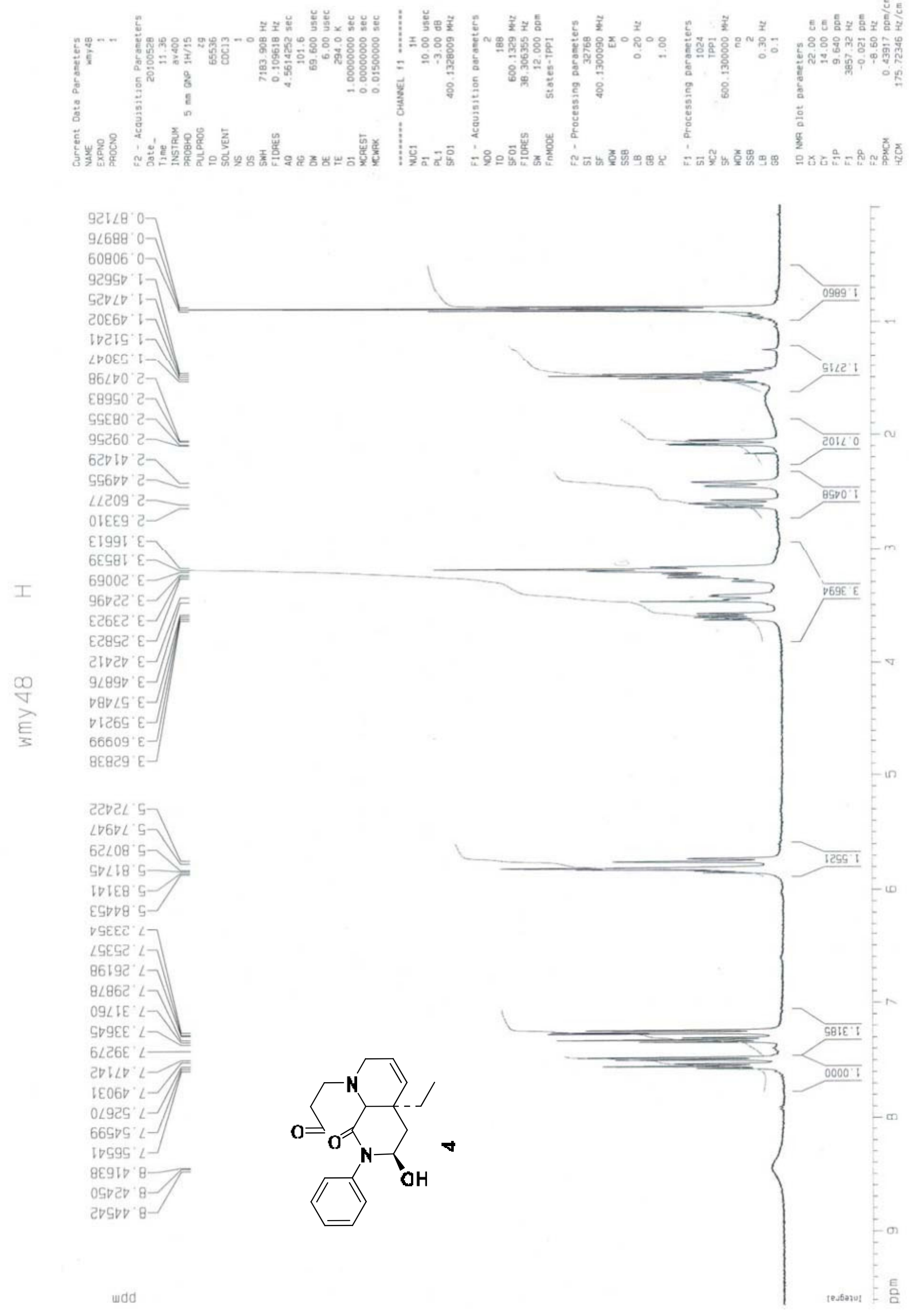

Figure 17. ^{13}C NMR spectrum of 14,15-dehydromelohenine B C (**4**)

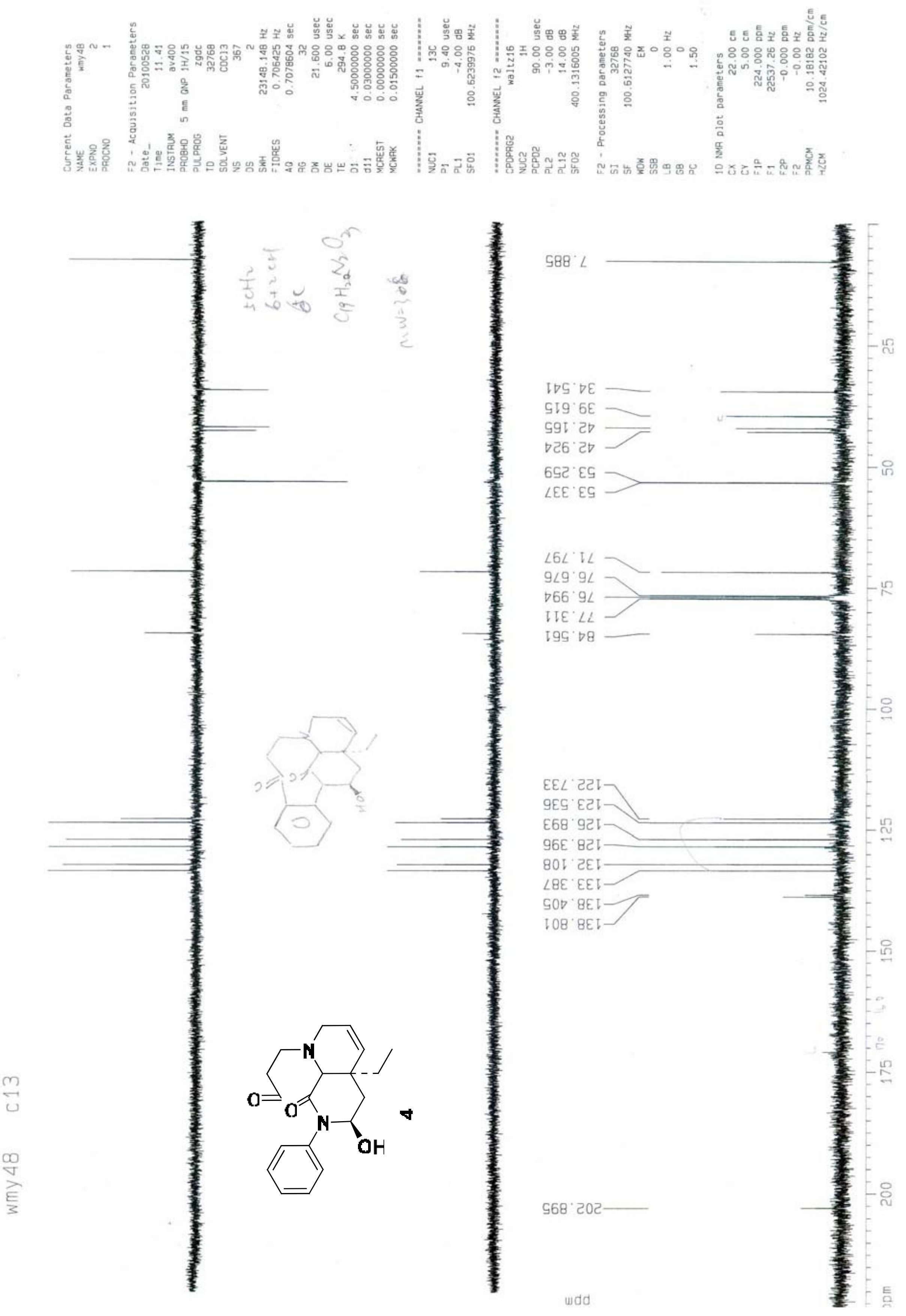

Figure 18. HSQC spectrum of 14,15-dehydromelohenine B (**4**)

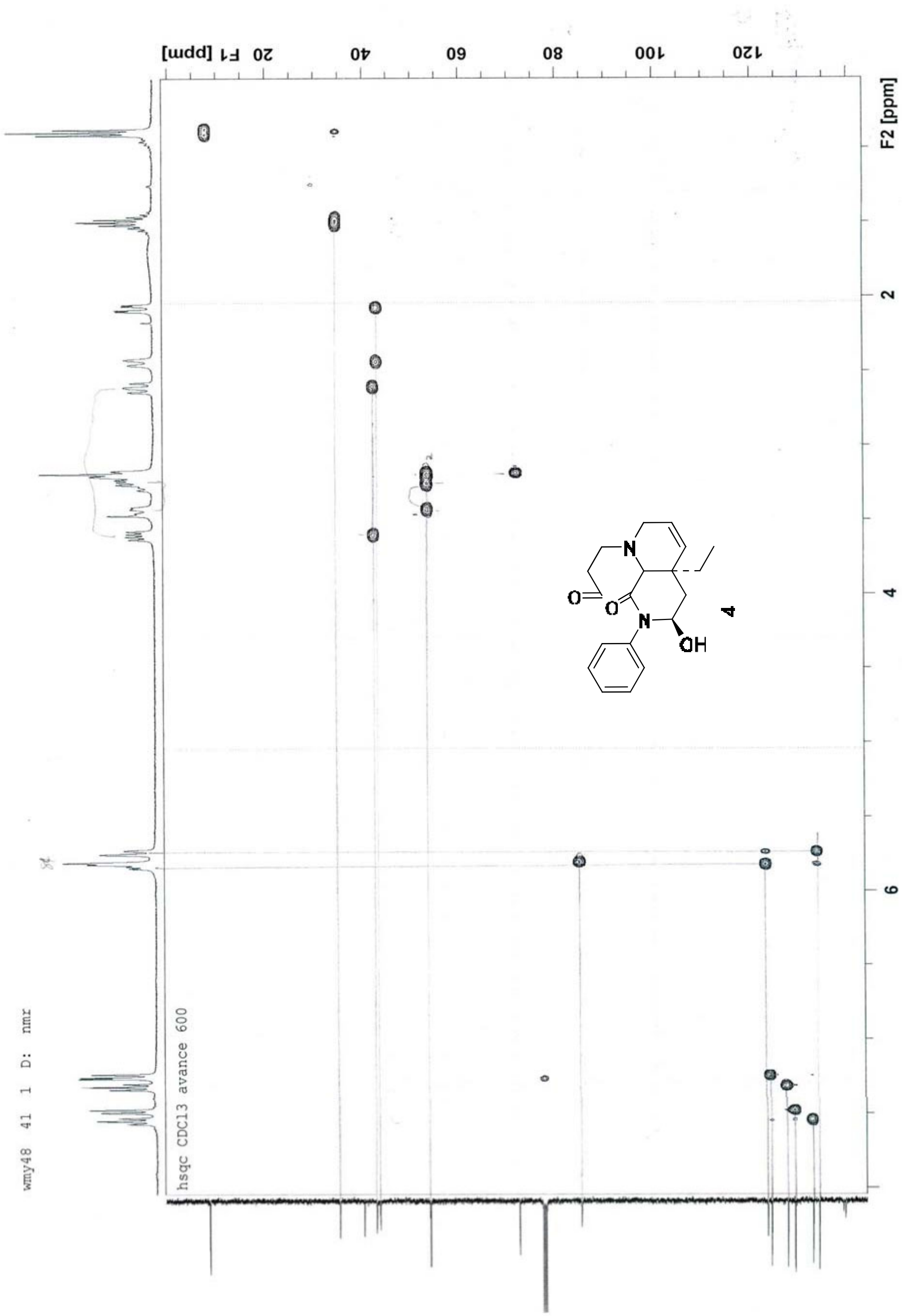

Figure 19. HMBC spectrum of 14,15-dehydromelohenine B (**4**)

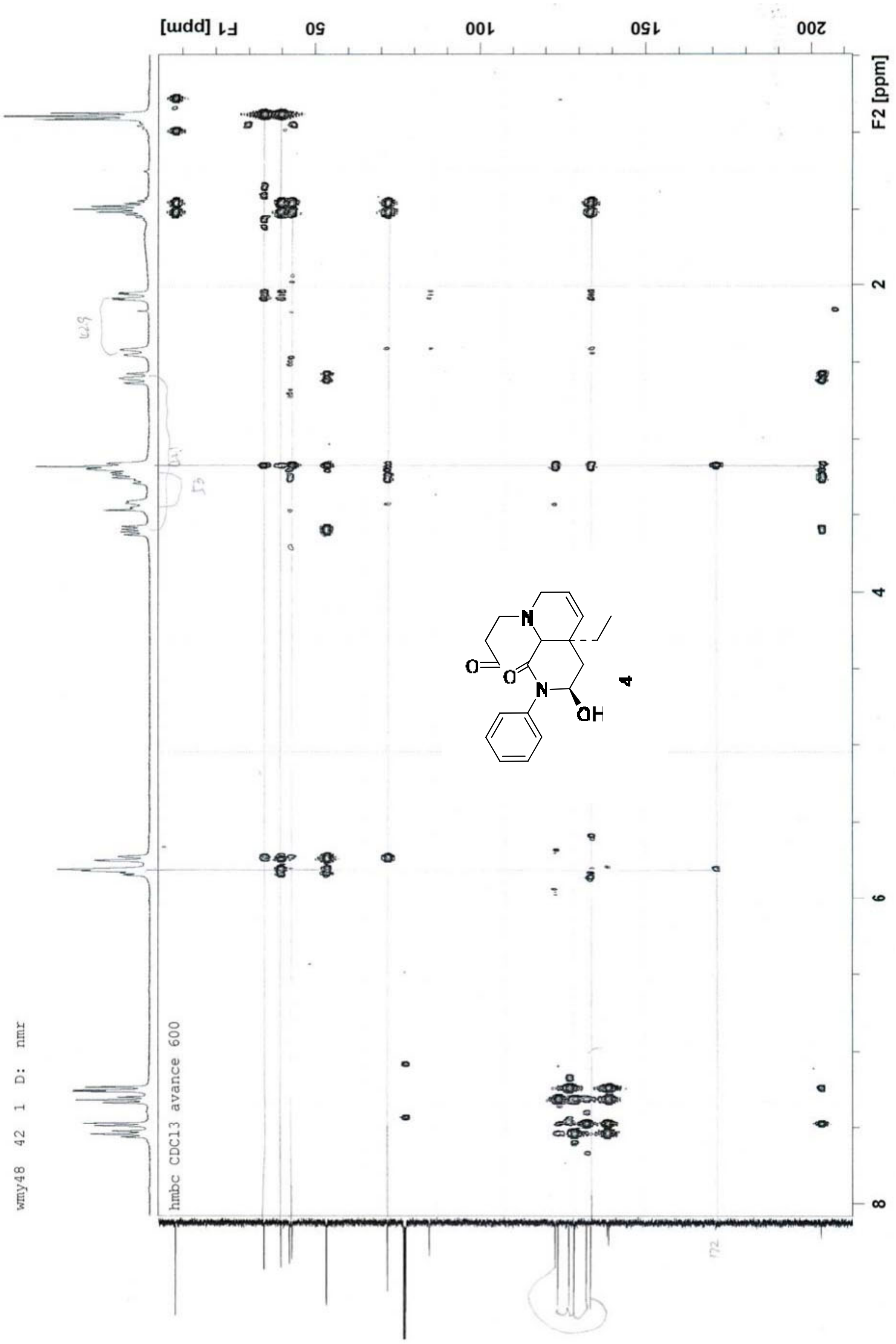

Figure 20. ROESY spectrum of 14,15-dehydromelohenine B (**4**)

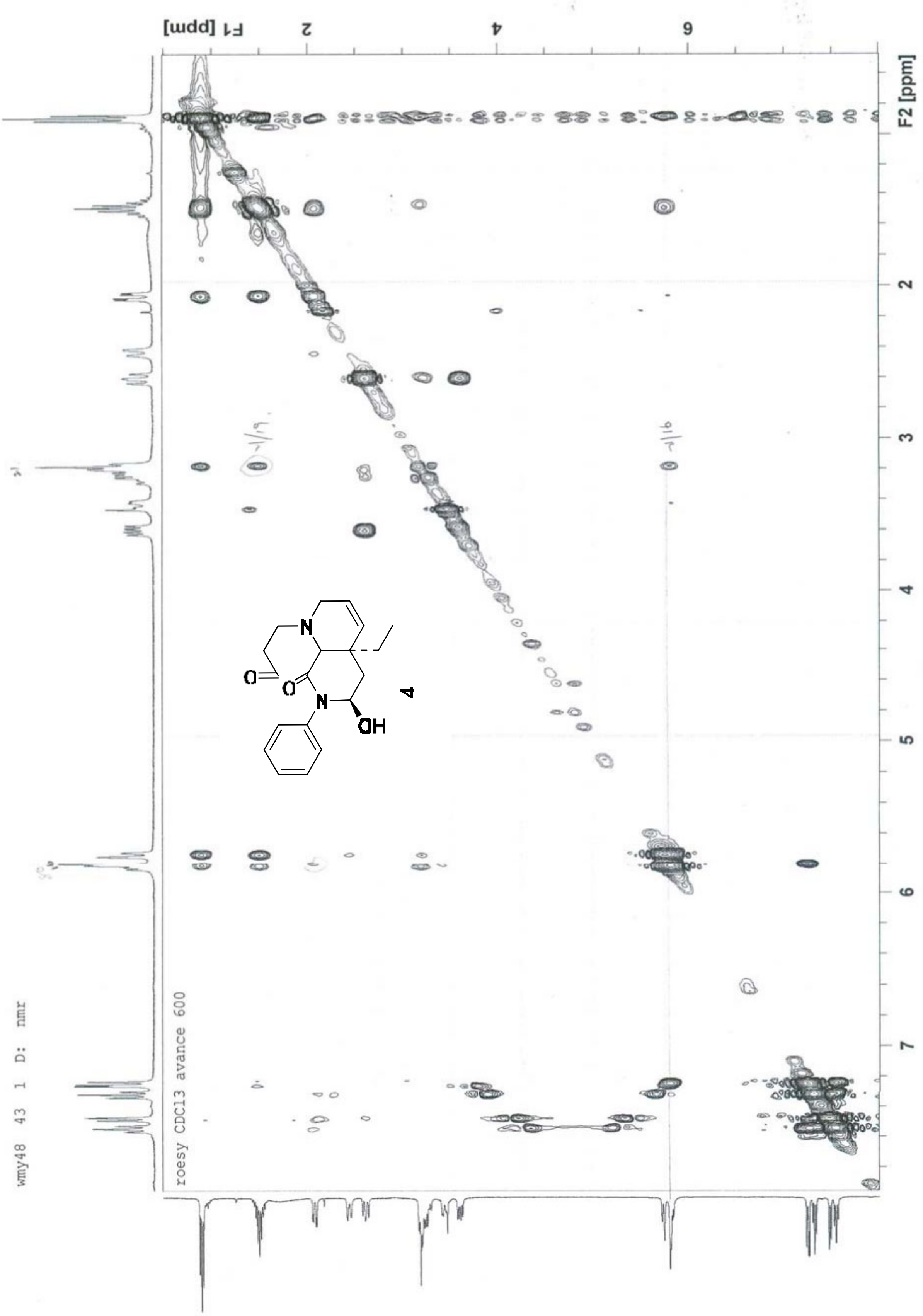

Supplement: Supplementary file 1 — Supplementary material, approximately 2.56 MB. [file 13659_2011_1_MOESM1_ESM.pdf]
